# From Oscillatory Transcranial Current Stimulation to Scalp EEG Changes: A Biophysical and Physiological Modeling Study

**DOI:** 10.1371/journal.pone.0057330

**Published:** 2013-02-28

**Authors:** Isabelle Merlet, Gwénaël Birot, Ricardo Salvador, Behnam Molaee-Ardekani, Abeye Mekonnen, Aureli Soria-Frish, Giulio Ruffini, Pedro C. Miranda, Fabrice Wendling

**Affiliations:** 1 INSERM, U1099, Rennes, France; 2 Université de Rennes 1, LTSI, Rennes, France; 3 Institute of Biophysics and Biomedical Engineering, Faculty of Science, University of Lisbon, Lisbon, Portugal; 4 Starlab Barcelona, Barcelona, Spain; 5 Neuroelectrics Barcelona, Barcelona, Spain; University College of London - Institute of Neurology, United Kingdom

## Abstract

Both biophysical and neurophysiological aspects need to be considered to assess the impact of electric fields induced by transcranial current stimulation (tCS) on the cerebral cortex and the subsequent effects occurring on scalp EEG. The objective of this work was to elaborate a global model allowing for the simulation of scalp EEG signals under tCS. In our integrated modeling approach, realistic meshes of the head tissues and of the stimulation electrodes were first built to map the generated electric field distribution on the cortical surface. Secondly, source activities at various cortical macro-regions were generated by means of a computational model of neuronal populations. The model parameters were adjusted so that populations generated an oscillating activity around 10 Hz resembling typical EEG alpha activity. In order to account for tCS effects and following current biophysical models, the calculated component of the electric field normal to the cortex was used to locally influence the activity of neuronal populations. Lastly, EEG under both spontaneous and tACS-stimulated (transcranial sinunoidal tCS from 4 to 16 Hz) brain activity was simulated at the level of scalp electrodes by solving the forward problem in the aforementioned realistic head model. Under the 10 Hz-tACS condition, a significant increase in alpha power occurred in simulated scalp EEG signals as compared to the no-stimulation condition. This increase involved most channels bilaterally, was more pronounced on posterior electrodes and was only significant for tACS frequencies from 8 to 12 Hz. The immediate effects of tACS in the model agreed with the post-tACS results previously reported in real subjects. Moreover, additional information was also brought by the model at other electrode positions or stimulation frequency. This suggests that our modeling approach can be used to compare, interpret and predict changes occurring on EEG with respect to parameters used in specific stimulation configurations.

## Introduction

Over the past 10 years, transcranial current stimulation (tCS) has emerged as a very popular tool for non-invasive brain stimulation as witnessed by its increasing use in the fields of cognitive neuroscience, neuropsychology and clinical neurology (for recent reviews see [1], [2]. It is now well established that tCS either with direct (tDCS) or alternating (tACS) current can induce both immediate and long-lasting effects on brain activity, in a polarity- and frequency-dependent manner.

To quantify these effects in humans, EEG is an ideal tool especially when stimulation is expected to interact with on-going brain rhythms. Recent human studies have shown that tACS applied at low frequency during sleep [3] or wakefulness [4] improved memory consolidation or encoding, while EEG delta activity was increased. In addition, an increase in EEG alpha power was reported after tACS was applied over the occipital cortex at individual alpha peak frequency [5]. Similarly, tACS applied transorbitally at alpha frequency in patients with partial blindness succeeded in restoring visual function and concomitantly increased the alpha power on EEG posterior electrodes [6].

Nevertheless, the relationship between changes in EEG and the biophysical and physiological aspects involved during tCS needs to be better understood. A first step is to determine the spatial distribution of the electric field generated in the brain when the transcranial induced currents flow through head tissues. This estimation is complex and relies on the physical and geometrical properties of the electrodes and of the different head tissues [7], [8]. Another step in understanding the effects of tCS relates to the impact of electric fields on the activity of neuronal populations within the cerebral cortex. These effects depend on many stimulation parameters such as the intensity or duration of the current, but also on the orientation of the pyramidal cells in the cortical layers [9], [10].

Computational models have recently brought insight into the manner in which electrical fields affect the activity of a neuronal assembly and its response, as observed in local field potentials (LFPs), and how these effects relate to the applied electric field parameters [11], [12]. The demand for integrating these aspects into a global modeling approach is high, as it would foster a link between tCS parameters and the effects of stimulation on cerebral activity.

In the present paper, we propose a model of EEG generation that integrates the effect of tCS on brain activity. Our modeling pipeline combines (1) the construction of physical models for current propagation in the brain in order to account for a realistic distribution of the electrical field on the cortex, (2) the use of neurophysiological mean field models to describe the local effect of applied currents on the activity of cortical populations of neurons (as reflected in local field potentials) and (3) the subsequent forward calculation in the realistic head model in order to reconstruct scalp EEG activity that reflects the global effects of tCS on brain neurodynamics.

After reporting the entire pipeline from stimulation configuration (head model, electrode size and position, current stimulation parameters) to generation of scalp EEG signals, we initially evaluate the face value of the model by attempting to reproduce the results of one of the studies mentioned earlier [5] and, secondly, question the predictive validity of our simulation approach.

## Materials and Methods

### Model of Neocortical Activity

#### Model description

In order to simulate signals generated in the cerebral cortex either spontaneously or under the influence of an externally-applied electrical field, we used a physiologically-plausible computational model recently developed in our team [12]. The level of modeling we used is that of the neuronal assembly. This model is composed of three subpopulations of cells (principal pyramidal cells and two types of interneurons) which interact via excitatory and inhibitory synaptic connections. The model accounts for the main types of cells present in the cerebral cortex, namely: i) Pyramidal cells which are identified as *type P* sub-population in the model, ii) Axon-, soma- and proximal dendrite-targeting cells (basket cells and chandelier cells) that mediate GABA_A,fast_ currents on *type P* cells and that are identified as *type I* interneurons, and iii) Dendrite-targeting cells (bitufted, bipolar and double bouquet cells) that mediate GABA_A,slow_ currents on *type P* cells and that are identified as *type I′* interneurons. Interactions between the three sub-populations are characterized by the connectivity constants 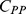, 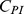, 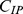, 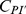, 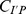, 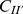, and 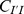 which account for the average number of synaptic contacts (or “connection strengths”) between sub-populations. The temporal dynamics of the model signal output directly compare with those reflected in real signals (local field potentials or LFPs) recorded with macro-electrodes located in the cerebral cortex.

**Figure 1:**
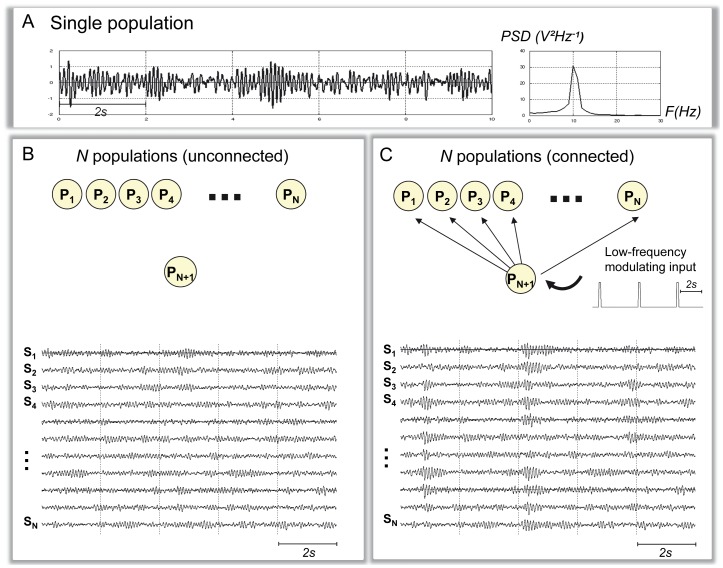
Generation of alpha-like cortical activity in the neuronal population model. **A:** Typical alpha-like signal (alpha peak around 10 Hz) produced by the model at the level of a single population for appropriate setting of parameters (see table 1) **B:** An example of signals obtained in the model with *N* populations (same set of parameters as in A) when no connectivity is present among populations. Alpha-like activity is generated at the level of each population. This activity is desynchronized among populations. **C:** An example of signals obtained in the model with *N* populations (same set of parameters as in A), when a “vertical” connectivity pattern is used. In that case, an additional population (*N+1^th^*) is added as a common synchronizer. This population called “subcortical” is unidirectionally coupled with the N other populations in order to mimic the thalamic input. The “sub-cortical” population also receives a direct low frequency input in order to mimic the synchronizing effect of cortical delta oscillations on the thalamus. Under these conditions, alpha-like activity is synchronized among the N populations.

Neuronal population models were originally developed to study the mechanisms underlying the generation of alpha activity in the cortex [13]. It is well-known that these models can easily reach an oscillating behaviour after appropriate setting of i) connectivity parameters and ii) rise and decay times of excitatory (glutamatergic) and inhibitory (GABAergic) average post-synaptic potentials (PSPs). In particular, at the level of a single population, when these parameters are properly adjusted, oscillations around 10 Hz become prominent in the model signal output [14]. These oscillations closely resemble real alpha activity (Figure 1A).

Model parameters used to simulate this alpha-like activity are provided in Table 1. Besides parameters controlling the kinetics of PSPs, the ratios between connectivity parameters (average number of synaptic contacts between sub-populations) were set according to physiology. In brief, the number of synaptic contacts among pyramidal cells and from pyramidal cells to interneurons was set to be greater than the number of synaptic contacts among interneurons and from interneurons to pyramidal cells. Readers may refer to appendix A of [15] for a literature review about the cellular organization of cerebral cortex with special emphasis on synaptic connections among pyramidal cells and interneurons.

**Table 1:** Parameters values by which the alpha like activity is obtained.

Synaptic gains	*A* = 5.5*B* = 8*G = *10
PSP rate constants	*a*_1_ = 40, *a*_2_ = 80*b*_1_ = 20, *b*_2_ = 60*g*_1_ = 150, *g*_2_ = 200 *s* ^−1^
Connectivity parameters	*C_PP_* = 55, *C_PI_* = 80, *C_PI’_* = 90*C_IP_* = 20, *C_II_* = 15*C_I’P_* = 25, *C_I’I_* = 20, *C_I’I’_* = 40
Wilson-Cowan sigmoids	*e*0*_P = _*10, *e*0*_I = _*10, *e*0*_I’_* = 10 *s* ^−1^ *v*0*_P = _*1, *v*0*_I = _*4, *v*0I’*_ = _*4 *mV* *r*0*_P = _*0.7, *r*0*_I = _*0.7, *r*0*_I’ = _*0.7 *mV^−1^*
tCS effect (gain ratios)	Pyramidal neurons: 1Fast interneurons: 0Slow interneurons: 0

As reported in [16], [17], a number *N* of neuronal populations can be coupled to model the activity over *N* interconnected brain structures. We followed this approach to obtain a “realistic” (i.e. relatively correlated) alpha-like activity over the entire neocortex (see Section 1.4 for practical details). Indeed, in the absence of any connectivity pattern, we could verify as expected that the *N* populations of the model generate uncorrelated alpha activity (Figure 1B) which is not compatible with real data. A way to overcome this difficulty is to account for the influence of the thalamic system which intervenes both in the generation and the control of alpha/spindles observed in EEG signals [18], [19]. It is noteworthy that our intent was not to develop a complex thalamo-cortical model but instead, to increase the “realism” of the simulated alpha-like neocortical activity. To proceed, in the model, we introduced an *N+1* ^th^ population, referred to as the “sub-cortical population”, that was unidirectionally connected to the *N* neocortical populations. Using this “vertical connectivity”pattern, the model could include a common input (mimicking, to some extent, the thalamic input) to neocortical populations. In order to also account for the periodicity of alpha bursts, a direct glutamatergic input was added to the sub-cortical population (periodic trapezoid pulse, 2.5 s period, i.e. 0.4 Hz). Under these conditions, we could qualitatively verify that the model generates “more realistic” alpha-like activity, as illustrated in Figure 1C.

#### Effect of tCS at neuronal population level

Neurons are sensitive to externally-applied electric fields in a geometry-dependent manner [10], [20], [21]. Their response depends on the orientation of the electric field with respect to the somato-dendritic axis on neuronal cells. More particularly, the field effects are maximal when the field orientation is parallel to the cells’ main axis. Conversely, field effects are null for orthogonal orientation. In addition to the orientation, it has been shown that the direction is also an essential factor. A field aligned with the orthodromic direction (dendritic tuft to axon) would result in a positive (depolarizing or excitatory) perturbation of the membrane potential at the soma. Conversely, a field in the antidromic direction would have a negative influence (hyperpolarizing or inhibitory). In accordance with these considerations, the influence of tCS was represented in the neuronal population model of as a perturbation on the mean membrane potential. Two main assumptions were made at this level. First, based on results reported in [10], it can be reasonably hypothesized that this perturbation is linear and direction-dependent within a certain range of magnitude. Second, we recently showed that the electric field induced by tCS has an impact on both pyramidal cells and interneurons [12]. Nevertheless, for the purpose of this study and in order to limit the number of free parameters, we neglected the field effects on interneurons since the impact is lower compared that on pyramidal cells [12].

Formally, we added a DC-offset voltage 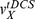 to the mean membrane potential 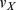 where 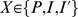. This DC-offset may have either a depolarizing or a hyperpolarizing effect on the sub-population of pyramidal cells, depending on its polarity. As a consequence, the firing rate of the corresponding sub-population described by the wave-to-pulse sigmoid function directly increases or decreases accordingly. It is worth mentioning that this effect on the firing rate is also consistent with results reported experimentally [10], as the applied field also modifies the action potential threshold.

### Physical Model for Current Propagation

In order to map the spatial distribution of the electric field on the cortical surface, we built a physical model for current propagation using a three-step approach.

**Figure 2:**
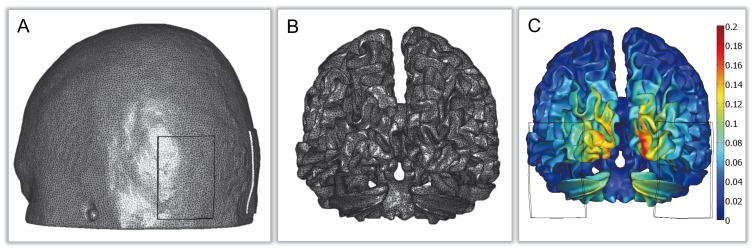
Physical model for current propagation. A: Finite element mesh of the scalp and of the stimulation electrodes **B:** Finite element mesh of the interface between GM and WM. **C:** Spatial distribution of the magnitude of the electric field, in V/m, induced by the injection of a current through the anode. In this stimulation configuration, the anode and cathode virtual electrodes are centered over both occipital cortices, and the amplitude of the injected current is set to 1.12 mA.

First MR images were segmented. For this purpose we used T1 and Proton Density (PD) phantom images based on the Colin27 template (http://www.bic.mni.mcgill.ca/brainweb). Segmentation was performed using the BrainSuite software (http://www.loni.ucla.edu/Software/BrainSuite, [22]–[25]). White matter (WM), gray matter (GM) and cerebrospinal fluid (CSF) were segmented from the T1 images whereas the skull and scalp were obtained from the PD images. The resulting masks were used to generate surface meshes representing the boundaries between the different tissues. The mesh of WM-GM interface had 189494 triangles and 94743 vertices (average area of triangles 1.036 mm2, maximum 3.832 mm2, minimum 0.008 mm2) and is shown in figure 2B. Secondly, a volume mesh representing the whole head was generated from the surface meshes using MIMICS (http://www.materialise.com/). At this stage, virtual tDCS electrodes were also incorporated into the model. The anode and the cathode could be placed at any of the positions defined in the 10–10 International System. The resultant mesh comprised more than 1.9×10^6^ tetrahedral second order Lagrange elements (Figure 2A). Thirdly, the volume mesh was imported into a finite element program to calculate the electric field (Comsol 3.5a, www.comsol.com). The upper surface of each electrode was set to uniform electrical potential and the potential difference between them was adjusted to reach the desired custom amplitude of injected current through the anode. All the remaining outer boundaries of the model were considered to be insulating, (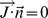), and continuity of the normal component of the current density was imposed on all the inner boundaries. The electric field induced in the brain was obtained by solving Laplace’s equation
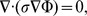
(where 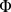 denotes the electrostatic scalar potential and 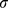 the electrical conductivity) and taking the gradient of the scalar potential. This procedure assumes that the quasi-static approximation [26] is valid for the low stimulation frequencies used in tCS. In this approximation the tissues are considered to be purely resistive with no capacitive components. The different tissues in the head model were modeled as having electrical conductivities with values close to the average ones reported in the literature for the DC/low frequency range [27]–[29]: 0.33 S/m, 0.008 S/m, 1.79 S/m, 0.33 S/m and 0.15 S/m for the scalp, skull, CSF, GM and WM, respectively. The electrodes were modeled as having a conductivity value arbitrarily taken to be equal to that of the scalp. All media were modeled as having isotropic conductivities and, as such, the current density could be found simply by multiplying the scalar electrical conductivity by the electric field.

All calculations were performed in Comsol, using its Conductive Media DC package. Given the large number of degrees of freedom in the model (more than 5×10^6^), an iterative linear system solver (GMRES) was chosen. This iterative solver required that a preconditioner be used (Incomplete LU with a drop tolerance of 0.005). The calculation took less than 3 hours to complete in a workstation with a quad-core i7–860 processor running at 2.8 GHz and 16 GB of RAM memory.

Following this calculation both normal and tangential components of the electric field were mapped on the WM mesh. The magnitude of the electric field on the WM surface is shown in (Figure 2C). The radial component of the electric field is discontinuous across the WM-GM boundary. Here we have always used the values obtained in the GM side of this interface.

### Generation of EEG Signals

EEG signals at 20 scalp electrodes (10–20 system) were simulated following our previous modeling approach [30]–[32]. In this model, EEG sources were represented as a dipole layer distributed over the cortical surface. The geometrical description of the cortical surface was achieved by using the same surface mesh (obtained from the segmented WM/GM interface) as the one used to map the electric field distribution. Each mesh triangle was associated to an elementary current dipole, placed at the barycentre of the triangle and oriented perpendicular to its surface. The magnitude of the moment of each dipole was proportional to the area of the corresponding triangle. In addition, each dipole was assumed to correspond to a distinct cortical neuronal population. Its time course, which represents the time-varying dynamics of the associated population, was provided by the output of a neuronal population model described in Section 1.1.

From this setup, we built a spatio-temporal source matrix 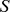 containing the time-varying activities of all cortical dipoles of the source space. Simulated EEG signals were generated using a realistic head model made of three nested surface meshes modeling the brain, the skull and the scalp with a conductivity of each medium fixed to 0.33 S/m, 0.008 S/m and 0.33 S/m, respectively [29]. The inter-medium surfaces were extracted from the segmentation of the same T1-weighted 3D-MRI as for the source space and meshed by 2440 triangles each (ASA, ANT, Netherlands). From this head model, the forward problem was then numerically calculated for each triangle using the boundary element method (BEM) implemented in ASA, to obtain the leadfield matrix 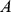. This matrix gives the contribution of each dipole of the mesh at the level of scalp electrodes. The spatio-temporal matrix 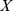 of simulated EEG data was given by
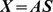



### Practical Implementation

**Figure 3:**
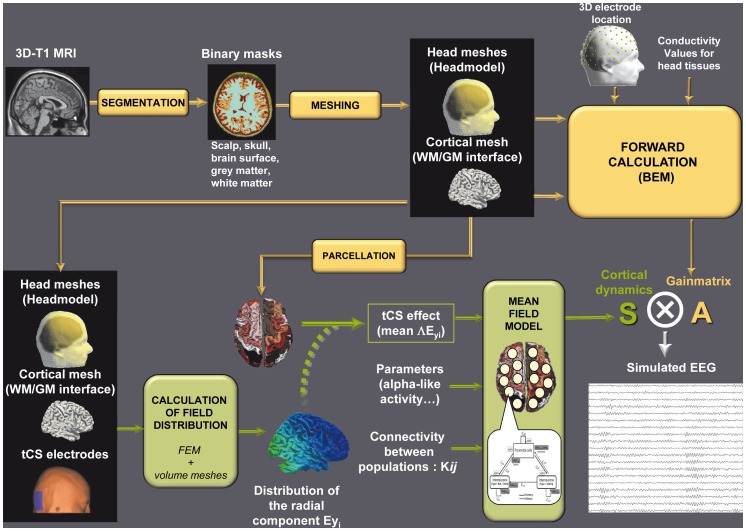
Simulation pipeline. 3D T1 MRI images are segmented into binary masks of the different head tissues in order to get meshes of the scalp, skull and brain surface (realistic head model) as well as of the white matter (WM)/grey matter (GM) interface. Unit dipoles are located at the barycenter of the triangles of this WM/GM mesh and set perpendicular to the triangle surface. This dipole layer over the cortex defines the source space. The forward problem is computed for each dipole using the Boundary Element Method (BEM) in order to get the leadfield matrix ***A*** that represents the contribution of each unit dipole of the mesh at each of the 19 scalp electrodes considered in our simulations (orange arrows in the pipeline). In order to get a physical model of the current distribution after tCS stimulation, surface meshes representing the boundaries between the different head tissues are transformed into volume meshes. In addition, virtual tCS electrodes are also represented into the model and can be placed at any scalp location (in our simulation protocol, we used PO9-PO10 location of the international 10–10 system). The electric field is calculated using the Finite Element Method (FEM) and the normal component of the field 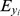 is mapped on the surface mesh of the WM/GM interface. 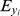 values are then averaged over 66 macro-regions to get the 66 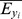 coefficients representing the mean field effect during tCS. We used then a model of coupled neuronal populations, with parameters of each population being adjusted to generate alpha-like activity, and connectivity between populations being defined in order to account for the thalamic input. 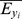 coefficients can be added to the average membrane potential of pyramidal cells of each cortical neuronal population in order to mimic the de- or hyper-polarizing effect of the electric field and to get the resulting time-varying activities at the level of each cortical macro-region (green arrows in the pipeline). The resulting spatio-temporal source matrix ***S*** is multiplied by leadfield matrix ***A*** to get the simulated EEG data under tCS condition.

In this section, we provide the reader with details about both the implementation of the model and the parameter values specifically used in this study. The proposed pipeline to simulate EEG data under tCS is summarized in Figure 3.

Theoretically, and according to the procedure described above, the time-course of the *N* dipole activities should be generated from a model of *N +1* coupled populations (N cortical +1 sub-cortical). As mentioned in sections 1.2 and 1.3, N = 189494 if one neuronal population is used per triangle of the mesh. In practice, the simulation of such a big network activity can hardly be achieved on standard PCs in a reasonable amount of time, given also that the duration of EEG signals to be simulated can be in the order of tens of seconds to minutes. Therefore, in order to reduce the numerical complexity and yet preserve the anatomical information, we regrouped the mesh triangles in 66 cortical regions (33 in each hemisphere) following the anatomical parcellation described in [33]. Contiguous triangles composing each of these regions were manually selected on the cortex mesh. The activity over each cortical macro-region was assumed to be homogenous. As described below, this assumption allowed us to represent the activity generated at the neocortex level by a network of 66+1 coupled neuronal populations.

To build the physical model for current propagation we considered two virtual electrodes (7×5 cm, thickness of approximately 0.3 cm) centred over the low occipital channels (PO9-PO10 locations). The amplitude of the injected current was set to 1.12 mA. This corresponded to the electrode configuration and mean current amplitude used in the reference study [5].

**Figure 4:**
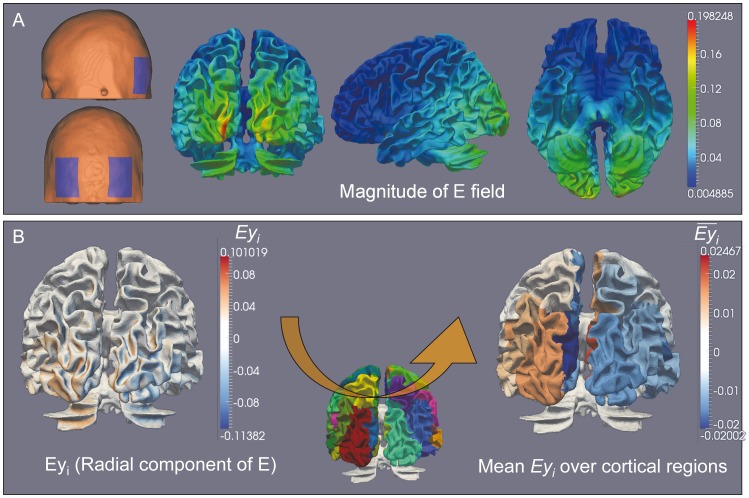
Spatial distribution of the externally-applied electric field and subsequent field effects. A: Spatial distribution of the magnitude of the electric field using two virtual electrodes over the occipital regions and after applying a 1.12 mA current through the anode. The amplitude of the electric field is mapped on a mesh of the WM/GM interface. **B:** Mean field effect. The effect of the electric field depending on the orientation of pyramidal cells within the neocortex, is accounted for by calculating the radial component of the applied field 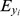 (scalar product between the electric field and the unit vector parallel to the pyramidal cell main axis) at each triangle of the mesh of the WM/GM interface. 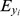 values obtained for each triangle are then averaged over each of the 66 anatomical macro-regions manually outlined according to [35]. The resulting 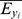 coefficients represent the mean effect of the electric field over each macro-region.

Using these parameters, the field distribution was calculated as described in section 1.2, and mapped on the WM surface mesh (representing the WM/GM interface) (Figure 4A). At each triangle of the mesh, the radial component of the externally-applied field (i.e. component parallel to the neuron main axis) 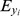, was obtained by
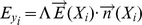
where 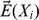 represents the electric field vector at the 3D position 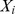, 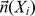 stands for the unit vector perpendicular to the triangle surface (i.e. parallel to the somatodendritic axis of pyramidal cells) and 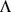 for a calibration constant.

The 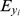 value accounted for the variable effect of the electric field component at a particular location over the neocortex surface (mesh triangle i) orientated along pyramidal cells. The maximal effect was obtained when 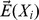 was aligned with the somatodendritic axis 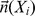 of pyramidal cells. 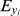 values obtained for each triangle were then averaged over each of the 66 macro-regions to obtain the 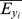 coefficients accounting for the mean effect of the electric field over each macro-region (Figure 4B).

In order to generate the cortical activity associated to each macro-region we used a model of 67 coupled populations, in which (1) the parameters of each population were adjusted to generate alpha-like activity and (2) the 67^th^ population accounted for the thalamic input as described in section 1.1. Each mean value 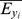 was added to the average membrane potential of pyramidal cells of each of the 66 cortical neuronal populations in order to get a set of 66 temporal dynamics. In order to calibrate the stimulation effect, 12 different sets of 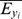 were considered depending on the 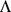 constant. 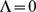 corresponded to the no-stimulation condition, and 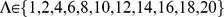 corresponded to the stimulation conditions. The alternating aspect of tACS was simulated by considering a sinusoidal stimulus waveform. Several tACS frequencies were tested (4 Hz to 16 Hz in steps of 1 Hz), with 10 Hz corresponding to the stimulation at the model alpha frequency. For the no-stimulation condition and for each frequency of the tACS condition, a set of 20 realizations of cortical activities were obtained.

In order to generate the scalp EEG signals we assigned all triangles within a given macro-region with the same time course and solved the forward problem as described in section 1.3. For each condition and simulation realization, at each scalp electrode, we calculated the mean signal power 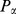 in the alpha frequency band as:
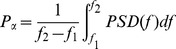



Where 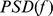 is the power spectral density of the signal at any considered electrode, 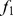 = 8 Hz, and 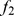 = 12 Hz. In practice, the *PSD* was estimated using the periodogram method on discrete simulated signals.

## Results

### Simulated EEG under no Stimulation Condition

**Figure 5:**
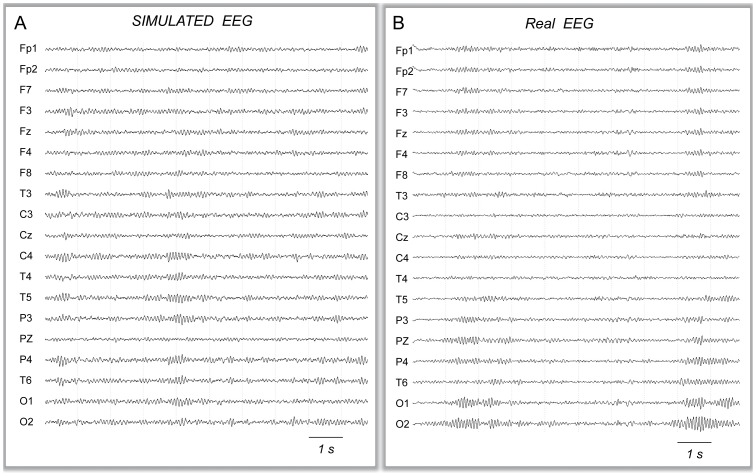
Simulation of EEG under no-stimulation condition. **A:** Typical signals simulated in the absence of tACS stimulation (19 scalp electrodes, international 10–20 standard system). **B:** Real alpha activity recorded in a normal subject during wakefulness with eyes closed.

An example of simulated EEG in the no-stimulation condition is displayed on Figure 5A. In the absence of tACS-like stimulation, simulated EEG signals display alpha-like activity. These rhythms are organised as spindles, synchronized over most of scalp EEG channels. This aspect is similar to that observed on real EEG alpha activity (Figure 5B). However, regarding real data, one can notice that the amplitude of the bursts of alpha activity is higher on parietal (P3, Pz, P4) and occipital channels (O1, O2) than on frontal electrodes while this specific topography does not appear on simulated data.

### Simulated EEG under tACS at Alpha Frequency (10 Hz)

**Figure 6:**
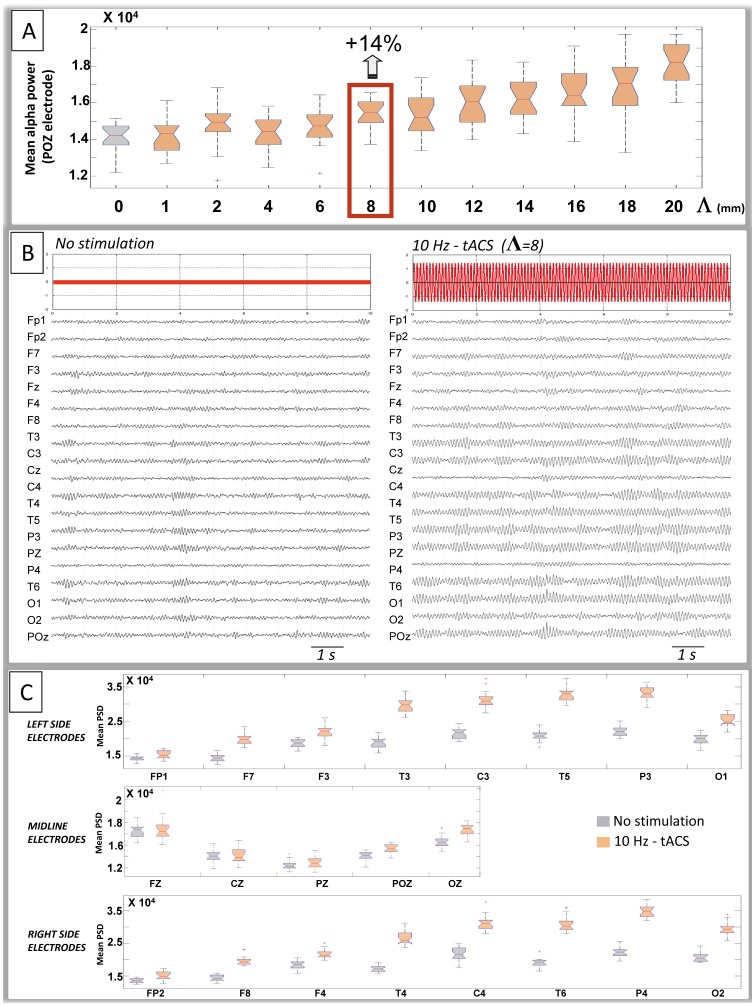
Simulation of scalp EEG signals under tACS stimulation. A: Calibration of the 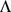 parameter. Scalp EEG signals were simulated for 13 values of parameter 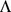 involved in the computation of the 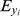 coefficients. 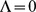 corresponds to the no stimulation condition, and 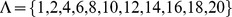 correspond to the 10 Hz-tACS stimulation condition. Boxplot are obtained after averaging the mean alpha power at the POZ scalp electrode from 20 trials of simulated EEG for each condition. During tACS stimulation the alpha power at posterior electrode POZ gradually increases with the calibration factor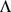. For 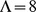, a significant increase of 14% is observed and corresponds to the percentage reported in real data at the same electrode [5]. This 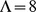 value was chosen for simulations. **B:** Typical simulated EEG signals obtained in the no stimulation (left) or during tACS at the model peak frequency (10 Hz). **C:** Mean alpha power, averaged from 20 trials at each of the 19 scalp electrodes in the no stimulation vs. 10 Hz-tACS condition. Significant increase in alpha PSD is observed during tACS stimulation at most left and right channels (except for fronto-polar electrodes) as well as at posterior midline channels POZ and OZ.

As mentioned in 1.4, the first step was to define the value of parameter 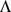. To proceed, we determined the value of 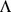 for which an increase of the signal power in the alpha frequency band at POZ similar to that reported in [5] was observed in the model. More specifically, when applied at the alpha-peak frequency of the model (10 Hz), the tACS-like stimulation induced an elevation of the alpha power at posterior electrode POZ. This elevation gradually increased when the calibration factor 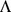 was augmented (Figure 6A). In the reference study [5], an elevation of 14% in the alpha power was observed at POZ electrode. In our simulation study, this percentage value corresponded to 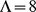. Therefore, the following simulations were calibrated by fixing 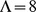.

At the level of all scalp electrodes, the increase in alpha power was clearly visible on left and right central, parietal temporal and occipital contacts, while only subtle changes occurred on midline electrodes (Figure 6B). As illustrated on (Figure 6C), the quantitative analysis revealed that under 10 Hz-tACS simulated stimulation, the alpha power was significantly increased on most left and right channels. The significant increase was larger on central and posterior channels and was maximal at the level of temporo-parietal electrodes (T5 or T6). Contrarily, the increase in alpha power remained non-significant at frontopolar contacts (Fp1, Fp2) and at the level of most anterior midline electrodes (Fz, Cz, and Pz).

### Simulated EEG under tACS at Other Frequencies

**Figure 7:**
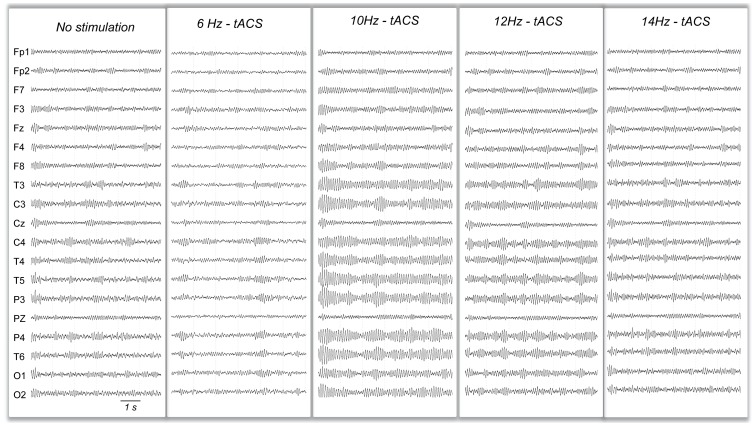
Example of simulated EEGs during variable frequency of tACS stimulation. A clear increase of the amplitude of alpha spindles is visible for 10 Hz- and 12 Hz- tACS but not for 6 Hz- or 14 Hz-tACS.

**Figure 8:**
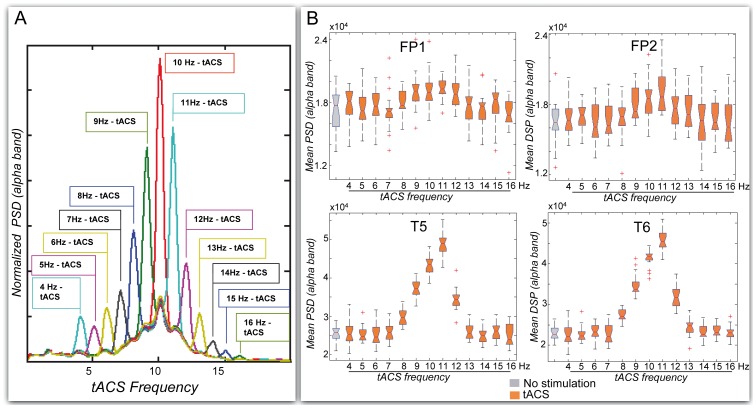
Effect of tACS stimulation frequency on EEG mean alpha power. A: Normalized alpha power obtained for a single epoch of simulated EEG and for variable tACS frequency (4 to 16 Hz). The maximal increase in alpha power is obtained for 10 Hz, i.e. at the alpha peak frequency of the model**. B:** Mean alpha power, averaged from 20 epochs of simulated EEG at variable tACS frequency. No significant increase occurs at fronto-polar electrodes at any tACS frequency. For all other channels, the increase in alpha power is significant only when tACS is applied at 8 to 12 Hz. Only T5 and T6 channels, displaying the maximal increase at these frequencies, are illustrated.

As compared to 10 Hz-tACS, 11 Hz and 12 Hz-tACS on the one hand, and 9 Hz and 8 Hz-tACS on the other hand had a progressively decreasing effect on alpha power density. An illustration is given for chosen frequencies on Figure 7. No significant effect was observed on simulated EEG when applying tACS at frequency outside of the [8 Hz–12 Hz] range (Figure 8).

## Discussion

The past years have witnessed significant advances in the use of tCS as a non-invasive tool for interacting with brain activity, not only in various brain disorders [34]–[38] but also during cognitive function [1], [39]. Among the many techniques that can be used to assess and quantify tCS effects, electroencephalography still occupies a central position, due to its excellent temporal resolution, reasonably good spatial resolution and relative ease of use. However, the interpretation of changes observed in EEG signals obtained under stimulation conditions is a challenging issue. In this study, a general modeling framework is put forward to progress in this interpretation. A complete pipeline is proposed to allow for the simulation of EEG signals based on i) a realistic description of the volume conductor features, ii) an accurate representation of the distribution of the electric field inside this volume conductor and iii) a model of the field effects on neuronal assemblies in the neocortex. To the best of our knowledge, the use of such a pipeline has not previously been proposed.

In order to evaluate the approach, we chose to start from a recent study conducted in humans that examined the effects of tACS on the alpha EEG activity [5]. This choice was motivated by both the low number of studies reporting quantified EEG changes in response to tACS and the relative ease with which oscillations in the alpha frequency band can be simulated with neural mass models. Using a physiologically-plausible neural mass model of coupled neocortical populations recently proposed by our team [12], we generated realistic signals of both cortical and related scalp EEG alpha activity. These signals were obtained using a very simple description of the interaction between neocortical macro-regions and a subcortical structure whose activity is simply described by a neuronal population model. Since the 70′s, this type of model has been widely used to reproduce physiological or pathological rhythms at the level of a single neuronal assembly [13], [40]–[42] or in a small networks of coupled assemblies [16], [43]–[46]. For simplicity reasons, we implemented an unsophisticated model for the thalamo-cortical interaction, as compared to those already available from the literature [47]–[49]. Indeed, we just used an extra neuronal population to provide common excitatory input to those accounting for neocortical activity. This helped pacemaking synchronized alpha oscillations over multiple cortical regions. Then, for the periodicity of alpha bursts, this sub-cortical population received itself a periodic excitatory input (periodic trapezoid “ramp up”, constant and “ramp down” function). One rationalization for this slow periodic excitatory input comes from findings that have emerged from the study of spindle oscillations during light sleep. Indeed, is has been shown, in the cat, that if the thalamus does not receive the slow wave cortical input, spindles are not “transmitted” to the cortex [50]. This mechanism of slow wave has been shown to be the mechanism responsible for synchronizing other brain activities in the brain at higher frequencies such as gamma activity [19]. Finally, studies in humans have also reported that slow EEG oscillations (below 1 Hz) during sleep modulated scalp EEG spindle activity [51]. Whether the same mechanism applies during wakefulness at resting state has not been shown. However, since very low frequency oscillations have been identified during rest in awake humans [52] and since alpha bursts resemble spindles, the synchronizing effect of slow oscillations on the alpha rhythm might reasonably be considered as a possible hypothesis.

In the present study, multiple populations are coupled in order to account for brain dynamics at a global level. Yet, our connectivity pattern is not sufficient to obtain a realistic topography of EEG alpha activity; in particular with respect to the parieto-occipital distribution of alpha waves during wakefulness. This can be explained by the fact that not only thalomo-cortical but also long-range cortico-cortical interactions are involved in the genesis of alpha activity [53]. Defining a connectivity pattern between cortical regions in our multiple population model is unfortunately a difficult task. While very interesting breakthroughs have been made in humans in particular with the use of Diffusion Tensor Imaging (DTI) data [54], it appears that directionality in the connectivity pattern, which is not yet available in humans, is essential for the realism of simulated dynamics in large-scale networks [55]. Recent work based on neuronal field models and dynamic causal modeling might be of interest to define connectivity patterns (both degree and direction) between cortical sources [56]–[58].

Despite the absence of cortico-cortical connectivity patterns, insights could be gained from our modeling approach into the effect of tACS stimulation on EEG alpha rhythms. Firstly, our modeled results are in accordance with the study by Zaehle and collaborators. Indeed, the increase in alpha power observed by these authors on posterior midline scalp electrodes (POz, Pz and Oz) was also reproduced in the model. In turn, the quantification of this increase on real data provided a method to calibrate the model. Interestingly, the value defined by the calibration process (Λ = 8) also corresponded to the first value of Λ for which significant changes occurred at the posterior electrode POz.

Secondly, beyond the results obtained in humans at posterior midline electrodes (POz, Pz, Oz), our modeling approach predicted changes at the level of electrodes that were not used in the EEG recordings by Zaehle et al. Indeed, the increase of alpha power in simulated EEGs was significant at most left and right electrodes, more prominently in the posterior than in the anterior and midline locations and maximal at the level of parietal and temporo-occipital channels. Interestingly, changes of maximal amplitude did not occur on occipital channels. This confirms that the effect of tCS is difficult to predict from the sole position (and area) of stimulating electrodes. In addition to the configuration of stimulating electrodes, the electric field spatial distribution and the mean orientation of cortical populations reached by the maximal field are crucial parameters upon which the topography of scalp EEG changes are dependent.

Thirdly, we did not observe any change in the power spectral density of alpha when stimulating the model outside the [8 Hz–12 Hz] frequency range, which precisely corresponds to the “individual peak frequency” (10 Hz) +/−2 Hz range described in humans by Zaehle et al. (2010). It is worth mentioning that the increase of power observed in simulated signals is not caused by the superposition of the EEG with the tACS stimulation artifact. Instead, it is caused by the fact that neuronal populations act as nonlinear dynamical systems which intrinsic response is more prominent when stimulated around their “natural” oscillation frequency (typically 10 Hz, here). In other words, in the model, the amplitude of alpha activity is enhanced when populations are stimulated at their resonance frequency. This result suggests that significant changes in power spectral density in a given frequency band occur when tACS frequency is set around that given frequency. This model-based hypothesis is indirectly confirmed by experimental data obtained in patients suffering from partial blindness and showing transorbital ACS at individual alpha frequency induces a significant increase of the EEG alpha power spectra at various scalp locations, including occipital channels [6]. Moreover, data related to the study of memory consolidation in healthy humans are also consistent with the model predictions as they show that 0.75 Hz –oscillatory tCS induces an increase in endogenous cortical slow oscillations both during slow wave sleep early stages [3] and during wakefulness [4]. The concomitant increase in theta activity observed in the latter study is left unpredicted in our simplistic model as it may relate to the complementary roles of theta and delta oscillations in the coupling of neocortical and hippocampal systems during learning and memory formation.

Finally, it is noteworthy that, as is, our modeling approach is focused on the instantaneous effects of oscillatory tCS on EEG activity while the above mentioned in vivo studies were devoted to post-effects on EEG [3]–[5]. However, it should be mentioned that these studies have considered immediate after-effects (i.e. immediately after tACS is switched off). Nevertheless, our results could certainly be further confirmed if the same stimulation protocol as in the present paper was reproduced in healthy controls in order to quantify instantaneous effects on EEG. At present, such an analysis remains difficult as the artifact generated by tACS is considerably masking recorded EEG signals. The removal of such an artifact, which dominates in the same frequency band as the expected changes of EEG rhythms, is a challenging issue that is currently being addressed in our group.
